# Nonpuerperal Breast Infection

**DOI:** 10.1155/S1064744995000330

**Published:** 1995

**Authors:** C. Miranda Casas, M. Pérez, J. C. Alados, J. Fontes, G. Orellana, J. M. Aguilar, J. A. Miranda, M. de la Rosa

**Affiliations:** 1 Microbiology Service Virgen de las Nieves Hospital Granada Spain; 2 Department of Gynecology and Obstetrics Virgen de las Nieves Hospital Granada Spain; 3 Servicio de Microbiologia HGE Virgen de las Nieves Avda. Coronel Muñoz, 2 Granada 18014 Spain

## Abstract

*Objective:* We undertook a microbiological study of purulent specimens from women with symptomatic breast abscesses.

*Methods:* Fifty-one purulent samples were collected in 2 periods (December 1991–April 1992 and January 1994–June 1994) from nonpuerperal breast abscesses in 44 patients attending our hospital.

*Results:* One of the most frequently isolated microorganisms was *Proteus mirabilis* (9 patients, 20.4%), present as a pure culture in all but 1 specimen (isolated together with *Peptostreptococcus* spp.). *Staphylococcus aureus* was isolated in 10 specimens, 6 of which were post-tumorectomy abscesses. Polymicrobial anaerobic flora were isolated in 11 specimens (21.5%); *Staphylococcus epidermidis* in 4 (8%); and *Streptococcus milleri,* *Alcaligenes* sp., and mixed aerobic-anaerobic flora in 1 specimen each. The 7 remaining samples (13.7%) were negative bacteriological cultures.

*Conclusions:* We draw attention to the frequent isolation of *P. mirabilis* in recurrent and torpid breast abscesses in 4 women in whom surgery was necessary in addition to antibiotic treatment.


Infectious Diseases in Obstetrics and Gynecology 3:64–66 (1995)
© 1995 Wiley-Liss, Inc.

### Nonpuerperal Breast Infection

**C. Miranda Casas, M. Pérez, J.C. Alados, J. Fontes, G. Orellana, J.M. Aguilar, J.A. Miranda, and M. de la Rosa**

*Microbiology Service (C.M.C., M.P., J.C.A., G.O., J.M.A., M.d.l.R.) and Department of Gynecology and Obstetrics (J.F., J.A.M.), Virgen de las Nieves Hospital, Granada, Spain*

### Abstract

***Objective:*** **We undertook a microbiological study of purulent specimens from women with symptomatic breast abscesses.**

***Methods:*** **Fifty-one purulent samples were collected in 2 periods (December 1991–April 1992 and January 1994–June 1994) from nonpuerperal breast abscesses in 44 patients attending our hospital.**

***Results:*** **One of the most frequently isolated microorganisms was *Proteus mirabilis* (9 patients, 20.4%), present as a pure culture in all but 1 specimen (isolated together with *Peptostreptococcus* spp.). *Staphylococcus aureus* was isolated in 10 specimens, 6 of which were post-tumorectomy abscesses. Polymicrobial anaerobic flora were isolated in 11 specimens (21.5%); *Staphylococcus epidermidis* in 4 (8%); and *Streptococcus milleri, Alcaligenes* sp., and mixed aerobic-anaerobic flora in 1 specimen each. The 7 remaining samples (13.7%) were negative bacteriological cultures.**

***Conclusions:*** **We draw attention to the frequent isolation of *P. mirabilis* in recurrent and torpid breast abscesses in 4 women in whom surgery was necessary in addition to antibiotic treatment.**
© 1995 Wiley-Liss, Inc.

Key words
Breast abscess, nonpuerperal mastitis, microbiology

*Staphylococcus aureus* has been recognized as the microorganism most frequently associated with breast abscesses. However, in the last few years, several studies have shown that other organisms, particularly anaerobes in pure and mixed cultures, are the most frequently found pathogens in nonpuerperal mastitis.[1–3]

Enterobacteria, mainly *Escherichia coli* and *Proteus* spp., have been isolated in all studies of nonpuerperal mastitis. Nevertheless, because they have been found less frequently than anaerobic organisms, they have not been considered etiologic agents of this disease when empirical antibiotic treatment has been planned.

In this study, we present the results of a microbiological study of specimens from women with symptomatic breast abscesses. The samples were received in our laboratory between December 1991 and April 1992 and between January 1994 and June 1994.

### MATERIALS AND METHODS

Fifty-one samples from 44 patients from the Breast Pathology Unit of the Gynaecology and Obstetrics Department at the Virgen de las Nieves Hospital, Granada, Spain, were analyzed. The samples were obtained during 2 periods: from December 1991 to April 1992 (period 1; 26 samples from 22 patients) and from January 1994 to June 1994 (period 2; 25 samples from 22 patients). The breast abscesses were classified on the basis of history and clinical presentation as acute (initial presentation of erythema with pain and tenderness or suppuration in the subareolar region), chronic or recurrent (2

Address correspondence/reprint requests to Dr. Consuelo Miranda Casas, Servicio de Microbiologia, HGE Virgen de las Nieves, Avda. Coronel Muñoz, 2, 18014 Granada, Spain.

Received June 21, 1994
Accepted February 27, 1995

or more episodes of recurrent infection by history associated with suppuration or draining sinus). The patients' ages ranged from 21 to 66 years.

All patients were diagnosed with nonpuerperal mastitis. In period 1, 7 of these patients were classified as having acute abscesses and 10 as having recurrent or chronic abscesses. The remaining 5 patients had abscesses that had appeared post-tumorectomy. The samples in the 1st period were obtained by fine-needle-aspiration biopsy (FNAB) (N = 11), surgical draining (N = 5), or spontaneous draining of the abscess (N = 10). In period 2, the abscesses in 7 patients were classified as acute, 7 as recurrent, and 8 as post-tumorectomy. The samples in the 2nd period were obtained by FNAB (N = 8), surgical draining (N = 9), or spontaneous draining (N = 8).

The samples were inoculated onto 5% horse blood agar (duplicate plates incubated in 5% $CO_2$ under anaerobic conditions), chocolate agar plates incubated in 5% $CO_2$, McConkey agar and mannitol agar incubated aerobically, and 5% horse blood agar with 100 μg/ml amikacin (LK) incubated under anaerobic conditions. The plates were incubated at 37°C and examined 24, 48, and 72 h after incubation.

Gram-stained preparations were made of all specimens to observe their suppuration and the presence of microorganisms. We considered significant the isolation of single or mixed potentially pathogenic microorganisms in specimens with abundant leukocytes and few or no epithelial cells. The specimens with epithelial cells and no leukocytes were not considered.

The isolates were identified using conventional diagnostic methods based on colony morphology, atmospheric growth conditions, and Gram staining.[4] Antibiograms were done on all isolates by the disk diffusion technique[5] or MIC determination by microdilution in automated PASCO broth (DIFCO, Detroit, MI).

### RESULTS

Seven of the 44 women had negative bacteriological cultures. Aerobic bacteria were isolated in 24 (64.8%) of the remaining 37 patients, polymicrobial anaerobic culture in 11 (39.7%), and mixed anaerobic and aerobic flora in 2.

In spite of the fact that duplicate specimens were obtained from 5 patients and a triplicate specimen from 1 (making a total of 51), the result of the second culture was the same as the first. *P. mirabilis* was isolated in all samples in pure culture in 4 patients; anaerobic mixed flora was found in both samples from the 5th patient; and *S. aureus* in both samples from the 6th patient.

The most frequent anaerobic isolates were *Peptostreptococcus anaerobius*, *P. assacharolyticus*, *P. magnus*, *Bacteroides* spp., and *Fusobacterium* spp. Nine (69.2%) patients with mixed anaerobic flora had recurrent breast abscesses.

In period 1, *Proteus mirabilis* was the most frequently isolated microorganism, being recovered in 7 (32%) patients, *Peptostreptococcus* sp. was found with *P. mirabilis* in a patient with a chronic breast abscess and as pure culture in 6 specimens, 3 of which belonged to patients with chronic or recurrent abscesses. In period 2, *P. mirabilis* was isolated in 2 patients, 1 with a recurrent abscess and another with an acute abscess.

*S. aureus* was isolated from 5 specimens in period 1 and 5 in period 2. These specimens were obtained from 6 post-tumorectomy breast abscesses, 3 acute breast abscesses, and 1 recurrent abscess.

*S. epidermidis* was the only isolate in 4 specimens from 3 patients with post-surgical breast abscesses and 1 with an acute abscess. We also found *Streptococcus milleri* in 1 woman with symptoms of recurrent abscess and *Alcaligenes* sp. in another woman with a post-tumorectomy breast abscess.

All *Staphylococcus aureus* isolates were sensitive to cloxacillin and resistant to penicillin and ampicillin.

Anaerobic microorganisms were sensitive to all of the antianaerobic antibiotics tested (metronidazole, amoxicillin-clavulanic acid, cefoxitin, and clindamycin).

*P. mirabilis* isolates were sensitive to most of the antibiotics tested (aminoglycosides; trimethoprim-sulfamethoxazole; first-, second-, and third-generation cephalosporins; amoxicillin; and amoxicillin-clavulanic acid). Only 3 isolates were resistant to amoxicillin and 3 to trimethoprim-sulfamethoxazole.

### DISCUSSION

Mild nonpuerperal breast abscess is often treated with antistaphylococcal agents.[6] In the last few years, many studies of the incidence of *S. aureus* in nonpuerperal breast abscesses have found that anaerobes rather than *S. aureus* are the microorganisms most frequently associated with this infection.[1,2,6] This finding is supported by our results: *S. aureus* was isolated in 10 of the 44 (22.7%) cases of breast abscess. However, 6 of these were post-surgical infections.

The high percentage of anaerobic microorganisms recovered in this study (13 patients, 29.5%) emphasizes their role as pathogenic agents in breast abscesses, although this percentage is less than that reported by other authors, who reported anaerobic recovery rates of 40–50%.[1,3,7]

According to Scholefield et al.,[7] nonpuerperal breast abscesses caused by anaerobic bacteria tend to cause intraductal stasis, which favors persistence of the infection. Some authors suggest that excision of the main galactophorous duct system would be an effective treatment.[8,9] Our results support this hypothesis, since 9 of the 13 cases of breast abscess with anaerobic flora relapsed and required extensive resection in addition to antibiotic treatment.

In our series, *P. mirabilis* was detected in a high proportion of patients (20.4%) and was the most frequent isolate in the first period studied (32%). This microorganism is also one of the most frequent Enterobacteria in the series reported by Edmiston et al.[6] and Brook,[2] although it represented a small proportion of the total number of isolates. To ensure that the frequent isolation of *P. mirabilis* in period 1 was not casual, we analyzed the etiology of nonpuerperal breast abscesses in 2 different periods. It is remarkable that *P. mirabilis,* although less frequent in the 2nd period, was the only Enterobacteria isolated in this infectious syndrome in the absence of a history of traumatic injury or previous surgery in the affected women, thus suggesting that it was a nosocomial infection.

Five of the 9 patients with *P. mirabilis* had severe recurrent and torpid chronic abscesses that were clinically similar to those caused by anaerobic bacteria. These lesions required extensive resection in addition to antibiotic therapy.

In the galactophorous ducts, *P. mirabilis* may have an effect similar to that in the urinary tract, where it causes stones by acting as a foreign body that maintains the infection.

It is worth noting that this species was isolated in pure culture in all but 1 case in which it was isolated together with *Peptostreptococcus* spp.

Since swarming strains of *Proteus mirabilis* can hinder the identification of small colonies or slow-growing anaerobes in nonselective media, we developed a routine method for reincubating LK plates for 72–96 h and inoculating plates containing another selective medium such as blood agar with nalidixic acid incubated anaerobically. This method prevents *P. mirabilis* growth and facilitates the recovery of possible anaerobic cocci in these samples.

Our results indicate that, in breast abscesses not associated with previous surgical procedures, *P. mirabilis* must be considered, together with anaerobic microorganisms, as one of the most frequent etiologic agents of nonpuerperal breast abscesses. Active antibiotic therapy against this bacteria should be included in empirical treatments.

### ACKNOWLEDGMENTS

We thank Karen Shashok for improving the English style of the manuscript.

### REFERENCES

1. Brook I: Microbiology of non-puerperal breast abscesses. J Infect Dis 157:377–379, 1988.
2. Brook I: The aerobic and anaerobic microbiology of neonatal breast abscess. Pediatr Infect Dis J 10:785–786, 1991.
3. Leach RD, Philips I, Eykyn SJ, Corrin B: Anaerobic subareolar breast abscess. Lancet 6:35–37, 1979.
4. Balows A, Hausler WJ, Herrmann KL, Isenberg HD, Shadomy HJ (eds): Manual of Clinical Microbiology. 5th ed. Washington, DC: American Society for Microbiology, 1991.
5. Barry AL, Thornsberry C: Susceptibility tests: Diffusion test procedures. In Balows A, Hausler WJ Jr, Herrmann KL, Isenberg HD, Shadomy HJ (eds): Manual of Clinical Microbiology. 5th ed. Washington, DC: American Society for Microbiology, pp 1117–1125, 1991.
6. Edmiston CE Jr, Walker AP, Krepel CJ, Gohr C: The nonpuerperal breast infection: Aerobic and anaerobic microbial recovery from acute and chronic disease. J Infect Dis 162:695–699, 1990.
7. Scholefield JH, Duncan JL, Rigers K: Review of a hospital experience of breast abscesses. Br J Surg 74:469–470, 1987.
8. Hadfield J: Excision of the major duct system for benign disease of the breast. Br J Surg 48:472–477, 1960.
9. Hughes L: *Bacteroides* and breast abscess. Lancet 2:198–199, 1976.
